# Supplementary material for: Validating a popular outpatient antibiotic database to reliably identify high prescribing physicians for patients 65 years of age and older
Source: PLoS One. 2019 Sep 26;14(9):e0223097. doi: 10.1371/journal.pone.0223097 (PMC6762161; doi:10.1371/journal.pone.0223097)
Supplement: S1 Table — (DOCX) [file pone.0223097.s001.docx]

| **Table S1:** Oral Outpatient Antibiotic Drugs Included in the Various Antibiotic Classes | |
| --- | --- |
| **Antibiotic Class** | **Antibiotic Drug Name** |
| Penicillin without Beta-lactamase Inhibitors | Amoxicillin  Ampicillin  Cloxacillin  Penicillin V potassium  Pivmecillinam |
| Penicillin with Beta-lactamase Inhibitors | Amoxicillin-clavulanate |
| First Generation Cephalosporins | Cefadroxil  Cephalexin |
| Second/Third Generation Cephalosporins | Cefaclor  Cefdinir  Cefixime  Cefpodoxime  Cefprozil  Ceftibuten  Cefuroxime |
| Second Generation Fluoroquinolones | Gemifloxacin  Ciprofloxacin  Norfloxacin  Ofloxacin |
| Third Generation Fluoroquinolones | Levofloxacin  Moxifloxacin |
| Macrolides | Azithromycin  Clarithromycin  Erythromycin  Spiramycin  Telithromycin |
| Trimethoprim and/or Sulphonamides | Sulfamethoxazole  Sulfamethoxazole-trimethoprim  Sulfisoxazole  Trimethoprim |
| Tetracyclines | Doxycycline  Minocycline  Tetracycline |
| Lincosomides | Clindamycin |
| Nitrofurantoin | Nitrofurantoin |
| Metronidazole | Metronidazole |
| Others | Rifabutin  Rifampin  Fidaxomicin  Fosfomycin  Linezolid  Methenamine  Tedizolid  Vancomycin |
