# Supplementary material for: Validating a popular outpatient antibiotic database to reliably identify high prescribing physicians for patients 65 years of age and older
Source: PLoS One. 2019 Sep 26;14(9):e0223097. doi: 10.1371/journal.pone.0223097 (PMC6762161; doi:10.1371/journal.pone.0223097)
Supplement: S1 Fig — Dash line = Mean difference (Xponent-ODB) = -0.5; Dotted lines = mean-2SD = -16.0 to mean+2SD = 15.0. (DOCX) [file pone.0223097.s002.docx]

Figure S1: Bland-Altman Plot from 9,272 physicians comparing the antibiotic rates (number of antibiotics prescribed per 100 total medications prescribed) between Xponent and Ontario Drug Benefit (ODB) for male patients only. Dash line = Mean difference (Xponent-ODB) = -0.5; Dotted lines = mean-2SD= -16.0 to mean+2SD = 15.0
