# Supplementary material for: Validating a popular outpatient antibiotic database to reliably identify high prescribing physicians for patients 65 years of age and older
Source: PLoS One. 2019 Sep 26;14(9):e0223097. doi: 10.1371/journal.pone.0223097 (PMC6762161; doi:10.1371/journal.pone.0223097)
Supplement: S2 Fig — Dash line = Mean difference (Xponent-ODB) = -0.1; Dotted lines = mean-2SD = -21.0 to mean+2SD = 20.7. (DOCX) [file pone.0223097.s003.docx]

Figure S2: Bland-Altman Plot from 9,272 physicians comparing the proportion of prolonged antibiotic duration (defined as >8 days) between Xponent and ODB for male patients only. Dash line = Mean difference (Xponent-ODB) = -0.1; Dotted lines = mean-2SD = -21.0 to mean+2SD = 20.7
