# Supplementary material for: Validating a popular outpatient antibiotic database to reliably identify high prescribing physicians for patients 65 years of age and older
Source: PLoS One. 2019 Sep 26;14(9):e0223097. doi: 10.1371/journal.pone.0223097 (PMC6762161; doi:10.1371/journal.pone.0223097)
Supplement: S3 Fig — Spearman correlation = 0.93; p<0.001. (DOCX) [file pone.0223097.s004.docx]

Figure S3: Correlation of antibiotic prescriptions per 100 total prescriptions in Xponent compared to antibiotic prescriptions per 100 patient visits in the Ontario Drug Benefit (ODB) database for male patients by primary care physicians. Spearman correlation = 0.93; p<0.001
